# Different feeding strategies can affect growth performance and rumen functions in Gangba sheep as revealed by integrated transcriptome and microbiome analyses

**DOI:** 10.3389/fmicb.2022.908326

**Published:** 2022-08-24

**Authors:** Zhang Jize, Deqing Zhuoga, Zhang Xiaoqing, Ta Na, Gesang Jiacuo, Luosang Cuicheng, Pingcuo Bandan

**Affiliations:** ^1^Institute of Grassland Research, Chinese Academy of Agricultural Sciences, Hohhot, China; ^2^Institute of Livestock Research, Tibet Academy of Agriculture and Animal Husbandry Science, Lhasa, China

**Keywords:** feeding strategies, Gangba sheep, growth performance, transcriptome, microbiome

## Abstract

Due to the harsh environment in the Tibetan Plateau, traditional grazing greatly limits the growth potential of local animals and causes severe ecosystem degradation. This is an urgent issue to be solved, which requires alternative strategies for grazing animals in the Tibetan alpine pastoral livestock systems. This study aimed to investigate the effects of different feeding strategies on growth performance and ruminal microbiota-host interactions in the local breed of sheep (Gangba sheep). Thirty 9-month old Gangba sheep (*n* = 10 per group) were assigned to natural grazing (G), semi-grazing with supplementation (T), and barn feeding (F) groups (supplementation of concentrate and oat hay) based on body weight. At the end of the experiment (75 d), all sheep were weighed, rumen fluid was obtained from six sheep per group, and ruminal epithelium was obtained from 3 sheep per group. The results showed that: (1) Compared with the G and T groups, the F group significantly increased dry matter intake, average daily gain, and feed conversion ratio of animals. Additionally, Gangba sheep in the F group had higher concentrations of ruminal short-chain volatile fatty acids (VFAs), especially propionate and butyrate (*P* <0.05) than sheep in the G and T groups. (2) The principal coordinates analysis indicated a significant difference in bacterial composition among different feed strategies. More specifically, the relative abundance of propionate (unidentified F082 and *Succiniclasticum*) and butyrate-producing (*Eubacterium_coprostanoligenes_group*) genera were also observed to be increased in the F group, in which unidentified F082 was identified as a differential biomarker among the three groups according to linear discriminant analysis effect size analysis. (3) The dynamics of the rumen epithelial transcriptome revealed that ECM-receptor interactions, focal adhesion, and PI3K-Akt signaling pathways, which are critical in mediating many aspects of cellular functions such as cell proliferation and motility, were upregulated in the F group. In conclusion, under harsh conditions in the Tibetan alpine meadow, barn feeding increased ruminal VFAs concentrations (especially propionate and butyrate), which stimulated gene expression related to cell proliferation in rumen epithelium, appearing to be superior to natural grazing and semi-grazing in gaining body weight of the local Gangba sheep.

## Introduction

As an abundant source of multiple essential nutrients, mutton is a valuable part of the diet for humans. Due to these health benefits, the worldwide demand for high-quality mutton has dramatically increased in recent years. The Gangba sheep is an important local sheep breed in Tibet, whose meat is famous for flavor and high nutritional value due to the geographical isolation and unpolluted environment in the Tibetan Plateau (Zhang et al., 2021). As natural grazing is still the primary feeding process of Gangba sheep (Zhang et al., 2021), the total number and individual productivity of sheep cannot be fully developed due to the fragility of the Tibetan alpine grassland ecosystem and harsh environmental conditions. Thus, there is an urgent need to improve high-quality mutton production while developing sustainable grassland ecosystems. In recent years, semi-grazing and barn feeding have become increasingly attractive as alternative strategies to replace traditional grazing. Numerous studies have demonstrated that these alternative feeding patterns improved the growth performance of several famous local sheep breeds (small-tailed Han sheep and Sunit sheep) in Inner Mongolia in China, which resulted in differences in gastrointestinal morphology and intestinal microflora, contributing to the gain of body weight and alternations of other crucial features of ruminants such as health status and even meat quality (Zhang et al., 2014, 2017; Wang B. et al., 2021; Wang Z. et al., 2021). To date, the underlying regulatory mechanisms for the Gangba sheep response to different feeding patterns have not been well-defined.

As a unique and important organ of ruminants, the rumen contains a dense and diverse microbiota, in which bacteria are the predominant domain for host feed digestion. These bacteria function synergistically to ferment plant materials, providing microbial proteins accounting for 90% of amino acids reaching the small intestine, as well as volatile fatty acids (VFAs) that are responsible for up to 80% of the metabolic energy supply (Liu et al., 2021). Among the various microbes in the rumen, cellulolytic microbes are the most prominent bacteria. Cellulolytic microbes secret cellulase and regulate the production of a variety of VFAs to provide bidirectional energy resources for ruminal epithelium development (Ouyang et al., 2019). In addition, the ruminal epithelium is crucial for multiple important physiological functions, such as nutrient absorption, transport, metabolism, and barrier function (Gäbel et al., 2002; Dai et al., 2017; Baaske et al., 2020). Previous studies also demonstrated a close relationship between the VFA profile and the gene expression patterns of gastrointestinal epithelial cells, which plays different physiological roles in ruminant and non-ruminant animals (Schlau et al., 2012; Sweeney et al., 2012; Liu L. et al., 2019). Thus, there is crosstalk between ruminal microbiota and the ruminal epithelium to maintain the natural metabolism and barrier function of the rumen (Lin et al., 2019).

However, little information is known about the effect of different feeding strategies on ruminal homeostasis, which is critical for the nutrient metabolism, growth, and health of ruminants (Penner et al., 2011) and could provide vital information to guide the breeding of Gangba sheep during the growth period. Here, we hypothesized that altered feed intake under different feeding regimes could cause variation in the Gangba sheep's growth performance through ruminal microbiota-host interactions. Hence, the current study was designed to explore the rumen fermentation parameters, the structure and function of ruminal bacterial communities, the transcriptome of the ruminal epithelium, and their interactions of Gangba sheep during growth period under different feeding patterns.

## Materials and methods

### Ethics statement

This study was carried out according to the Regulations for the Administration of Affairs Concerning Experimental Animals of the State Council of the People's Republic of China. The research protocol was approved by the Committee on Experimental Animal Management of the Chinese Academy of Agricultural Sciences (Beijing).

### Animals and treatments

The experiment was conducted at a local village-owned farm in Kamba County (88°25′E, 27°59′N, with an average altitude of 4,700 m), Tibet Autonomous Region, China, from July to October 2020. Thirty 9-month old Gangba sheep with similar body weights (14.08 ± 0.79 kg) were selected and randomly divided into three groups (*n* = 10 per group): natural grazing (G), semi-grazing with supplementation (T), and barn feeding (F). All Gangba sheep were purchased from the local farm where sheep traditionally graze on native pasture without concentrate supplementation. After marking with ear tags, the sheep were immunized and parasites expelled, and a 10-day period of adaptation followed by 75 days of different feeding regimens was implemented. The sheep in the G group were grazed on pasture without any supplementation and housing. The pasture of the grazing site was dominated by *Artemisia minor, Iris collettii, Festuca wallichanica, Kobresia deasyi*, and *Kobresia capillifolia*.

The sheep in the T group were grazed under the same environmental conditions as mentioned above in the morning (from 10:00 to 15:00 h) and housed in individual pens supplemented with 200–300 g concentrate and free access to oat hay when off pasture. The sheep in the F group were confined to a dry barn in individual pens with daily rations (400–500 g concentrate and free access to oat hay). The supplementation of concentrate and oat hay in the T and F groups were increased along with increasing body weight. The supplemented oat hay was adjusted daily based on the previous day's intake, allowing refusals of 20%. The leftover hay was weighed daily for individual sheep. Pasture dry matter intake (DMI) of the G and T groups was estimated using the *n*-alkane method in early August, September and October; details of this procedure were described in Mayes and Dove (2000). Water was provided *ad libitum* to the Gangba sheep during the experimental period. The daily ration was designed according to the Chinese Feed Standard of Meat-Producing Sheep and Goats (NY-T816-2004) (Ministry of Agriculture and Rural Affairs of the People's Republic of China, 2004). The chemical compositions of natural pasture, oat hay and supplemented concentrate are shown in Table 1.

**Table 1:** Nutrient composition of the concentrate, oat hay, and natural pasture (% of dry matter).

**Nutrient composition**	**Concentrate^a^**	**Oat hay**	**Pasture**
Dry matter (105°C)	88.30	90.03	89.99
Metabolizable energy, MJ/kg	12.08	9.92	–
Crude protein	16.10	10.06	8.93
Neutral detergent fiber	8.68	52.89	59.82
Acid detergent fiber	6.01	28.72	48.01
Calcium	0.54	0.40	2.27
Phosphorus	0.38	0.27	0.12

a The concentrate consisted of 55% chopped maize, 10% highland barley, 23.5% pea, 10% wheat bran, 1% limestone and 0.5% premix. The premix was formulated to provide (per kilogram of dry matter) vitamin A, 10,500 IU; vitamin D3, 2,110 IU; vitamin E, 43 mg; Mn, 40 mg; Fe, 32 mg; Zn, 95 mg; Cu, 16 mg.

Metabolizable energy was calculated value.

### Sample collection and analysis

The natural pasture collection procedure, as well as analyses of the natural pasture, concentrate and oat hay were described in a previous study (Ren et al., 2019). For those samples, dry matter (DM) content was determined according to AOAC (Association of Official Analytical Chemists) (1990). Nitrogen (N) was measured using a Kjeldahl analyzer (Kjeltec 2300, Hoganas, Sweden), and crude protein (CP) was calculated as 6.25 × N. Neutral detergent fiber (NDF) and acid detergent fiber (ADF) were determined by the filter bag technique (ANKOM 2000, Fairport, NY). *N*-alkane content of pasture was measured according to the modified procedure described in (Lin et al., 2006).

At the end of the experiment, all sheep were fasted for ~12 h and weighed. Six sheep of each group with similar weights to the average group weight were transported to a commercial slaughterhouse and sacrificed by CO_2_ asphyxiation. The epithelium samples of the dorsal rumen (about 1 g) were collected from sheep for transcriptomic (*n* = 3) and reverse-transcription quantitative PCR (RT-qPCR; *n* = 6) analyses. The rumen fluid contents were collected from individual sheep and then filtered through four layers of cheesecloth cloth. All collected samples were snap-frozen in liquid nitrogen and stored at −80°C until analysis. Approximately 2 ml of additional rumen fluid sample for each sheep was collected for 16S rRNA sequencing. Another 5 ml of rumen fluid sample for each sheep was collected for analysis of VFA profiles (gas chromatography, Agilent 6850, Agilent Technologies Inc., Santa Clara, CA, USA) and NH_3_-N concentrations (Xue et al., 2017).

### Transcriptome analysis of rumen epithelium

Total RNA of ruminal epithelium samples for RNA-Seq analysis was extracted using a Trizol reagent kit (Invitrogen, Carlsbad, CA, USA) according to the manufacturer's protocol. RNA quality was assessed on an Agilent 2100 Bioanalyzer (Agilent Technologies, Palo Alto, CA, USA) and checked using RNase-free agarose gel electrophoresis. Only samples that had an RNA integrity number (RIN) >7.0 were used for sequencing. The sequencing data were deposited in the Sequence Read Archive of the National Center for Biotechnology Information database with accession project number PRJNA761328.

All nine sequencing libraries were constructed according to the Illumina^®^ TruSeq^TM^ RNA sample preparation protocol. Then, paired-end sequencing (2 ×125 bp) was performed using Illumina HiSeq 2500 by Gene Denovo Biotechnology Co. (Guangzhou, China). High-quality clean reads were obtained by filtering using fastp (version 0.18.0) with following parameters: (1) removing reads containing adapters, (2) removing reads containing more than 10% unknown nucleotides (N), and (3) removing low quality reads containing more than 50% low quality (*Q*-value ≤ 20) bases. The index of the reference genome was built using Bowtie2 (version 2.2.8) (Langmead and Salzberg, 2012), and sequences were aligned to the sheep genome (*Ovis aries*, Ensembl_release 96) using HISAT 2.2.4. StringTie software was used to calculate the fragments per kilobase of transcript per million mapped reads (FPKM) to quantify the gene expression abundance and variations. DESeq2 software was used to screen differentially expressed genes (DEGs) between two groups. Genes with a false discovery rate (FDR) below 0.05 and absolute fold change ≥2 were identified as DEGs. Finally, the Kyoto Encyclopedia of Genes and Genomes (KEGG) was used to conduct pathway enrichment analysis of DEGs using KOBAS 2.0 software (Ren et al., 2019).

### Real-time PCR (qRT-PCR) analysis of genes in rumen epithelium

cDNA was reverse transcribed from extracted total RNA using a cDNA Synthesis Kit (Takara, Dalian, China). The expression of selected target genes (*n* = 6 per group) was determined via qRT-PCR on a QuantStudio 5 Real-time PCR Instrument (Applied Biosystems, Foster, CA, USA) with a SYBR Green Kit (Takara, Dalian, China) under the standard program. The relative expression of genes was normalized to the housekeeping gene (β-actin, *ACTB*) using the 2^−Δ*ΔCt*^ method (Ren et al., 2019). Specific primer sequences are shown in Supplementary Table 1.

### Rumen bacterial DNA extraction and high-throughput sequencing

Microbial DNA was prepared and extracted from each of the rumen fluid samples using the HiPure Stool DNA Kit (Angen, Guangzhou, China). The integrity of the DNA was assessed using 1% agarose gel electrophoresis, and the quality and concentration of each DNA sample were measured on the Nanodrop spectrophotometer (Thermo Scientific, Madison, WI, USA). Individual amplicon libraries were prepared by PCR amplification of the V3-V4 region of the 16S rRNA using the primer set 341F (5′-CCTACGGGNGGCWGCAG-3′) and 806R (5′-GGACTACHVGGGTATCTAAT-3′) with barcodes (Cui et al., 2020). Amplicons were extracted from 2% agarose gels, purified using the AMPure XP kit (Beckman, Indianapolis, IN, USA) according to the manufacturer's instructions, and quantified using the ABI StepOnePlus Real-Time PCR System (Life Technologies, Foster City, CA, USA). Purified amplicons were pooled in equimolar amounts and paired-end sequenced (2 ×250 bp) on an Illumina HiSeq 2500 platform according to standard protocols. Paired-end clean reads from the original DNA fragments were merged as raw tags using FLASH with a minimum overlap of 10 bp and mismatch error rates of 20%. Reads were subsequently filtered by QIIME quality filters. Then, the clean tags were clustered into OTUs of ≥97% similarity using UPARSE (version 9.2.64) (Edgar, 2013), and chimeric sequences were identified using UCHIME (Edgar, 2010). The representative OTU sequences were taxonomically classified into organisms by a naive Bayesian model using RDP classifier based on the SILVA database (Caporaso et al., 2010) with the confidence threshold value of 0.8. Then OTUs were normalized to the relative abundance in each sample before further analysis using the “otutab_rare” function of USEARCH. The alpha diversities, including Chao1 and Shannon indices, were analyzed to estimate the bacterial diversities using the Kruskal–Wallis test with multiple testing corrections (False Discovery Rate, FDR). Beta diversity analysis was carried out using unweighted UniFrac distance metrics and principal coordinates analysis (PCoA) to estimate differences in bacterial communities between samples. The microbiota biomarker feature in each group was screened by linear discriminant analysis (LDA) effect size (LEfSe) analysis based on the Kruskal–Wallis test. The *P*-value for significance was set as <0.05, and the threshold of the LDA score was set at a default value of 2.0, for which the relative abundance was significantly different among groups (LDA value > 4).

### Statistical analysis

Total DMI and average daily gain (ADG) of Gangba sheep in each treatment from August to October were analyzed using repeated measures in the MIXED procedure of SAS (SAS Institute Inc., version 9.2, USA). The following model was used: Y_jki_ = μ + β_j_ +A_k_ + B_i_ + AB_ki_ + e_jki_, where Y_jki_ is the target variable, μ is the overall mean, β_j_ is the random effect of an individual animal, A_k_ is the fixed effect of the treatment, B_i_ is the fixed effect of the month, AB_ki_ is the interaction effect of the treatment × month, and e_jki_ is the residual error. Differences among means of treatments were compared by Duncan's multiple range test. The differences related to rumen fermentation, mRNA expression, and bacterial abundance among groups were analyzed using one-way ANOVA followed by Duncan's *post hoc* testing in SAS v. 9.2. Results were presented as mean ± SEM. Differences were considered statistically significant at *P* <0.05. The correlation between rumen VFA concentrations and bacteria abundance was performed using Spearman's correlation test in SAS v 9.2, where *P* <0.05 and an absolute value of correlation coefficient *r* > 0.8 were regarded as significant correlations.

## Results

### Growth performance

Significant differences in growth performance of Gangba sheep among the treatments were identified, where the increased intake was found for sheep on both concentrate supplementation groups (Table 2). Compared with sheep in the G and T groups, sheep in the F group exhibited significantly increased ADG (Table 2).

**Table 2:** Effects of different feeding strategies on the growth performance of Gangba sheep.

**Item**	**Feeding strategy** ^**1**^			**SEM**	***P*-value**
	**G**	**T**	**F**		
Initial weight, kg	13.88	14.17	14.21	0.185	0.795
Final weight, kg	22.02^b^	24.69^a^	26.41^a^	0.616	0.005
Average daily gain, g/day	108.53^c^	140.28^b^	162.78^a^	7.300	0.003
Dry matter intake, kg/day	1.26^b^	1.45^ab^	1.52^a^	0.082	0.042
Feed conversion ratio	11.7^a^	10.5^b^	9.1^c^	0.625	0.037

^a, b, c^ Values in the same row with different superscript letters are statistically different at P <0.05.

Dry matter intake = Concentrate dry matter intake + pasture dry matter intake + oat hay dry matter intake, and pasture dry matter intake was estimated for T and F groups using the n-alkane method in early of August, September and October.

^1^G, natural grazing; T, semi-grazing with supplementation; F, barn feeding.

### Rumen fermentation profiles

Significant alternations of rumen fermentation profiles were observed among the three groups under different feeding strategies (Table 3). Gangba sheep in the G group had the highest pH value (*P* <0.05). The concentrations of NH_3_-N, acetate, and isovalerate in the T and F groups were higher (*P* <0.05) than in the G group. The concentrations of total volatile fatty acid (TVFA), propionate, butyrate, isobutyrate, and valerate in the F group were higher (*P* <0.05) than in the G and T groups, while acetate:propionate in the F group was the lowest compared with the G and T groups (*P* <0.05).

**Table 3:** Effects of different feeding strategies on ruminal fermentation in Gangba sheep.

**Item**	**Feeding strategy** ^**1**^			**SEM**	***P*-value**
	**G**	**T**	**F**		
NH_3_-N (mg/dl)	8.57^b^	21.48^a^	23.48^a^	2.37	<0.001
pH	6.85^a^	6.64^ab^	6.47^b^	0.068	0.025
TVFA (mmol/L)	38.4^c^	46.7^b^	55.7^a^	3.5	0.012
Acetate (mmol/L)	28.79^b^	35.17^a^	39.61^a^	2.32	0.027
Propionate (mmol/L)	6.33^b^	6.67^b^	10.06^a^	0.75	0.049
Acetate:propionate	4.55^a^	5.28^a^	3.94^b^	0.25	0.042
Butyrate (mmol/L)	1.85^b^	2.00^b^	2.36^a^	0.09	0.014
Isobutyrate (mmol/L)	0.48^c^	1.04^b^	1.35^a^	0.13	<0.001
Valerate (mmol/L)	0.19^b^	0.43^b^	0.63^a^	0.07	0.014
Isovalerate (mmol/L)	0.72^b^	1.47^a^	1.73^a^	0.17	0.015

^1^G, natural grazing; T, semi-grazing with supplementation; F, barn feeding; SEM, standard error of means; TVFA, total volatile fatty acid.

^a, b, c^Values in the same row with different superscript letters are statistically different at P <0.05.

### Rumen bacterial communities

**Figure 1:**
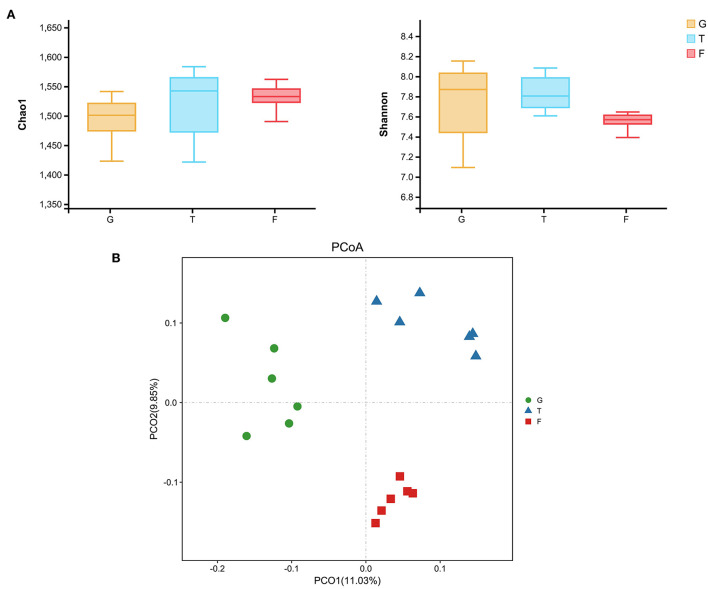
Effects of feeding strategies on ruminal microbiota of Gangba sheep. **(A)** The richness and diversity indices of rumen microbiota in Gangba sheep under different feeding strategies. **(B)** Principal coordinates analysis (PCoA) of the overall rumen microbiota in Gangba sheep based on unweighted UniFrac distance. G, natural grazing; T, semi-grazing with supplementation; F, barn feeding.

**Figure 2:**
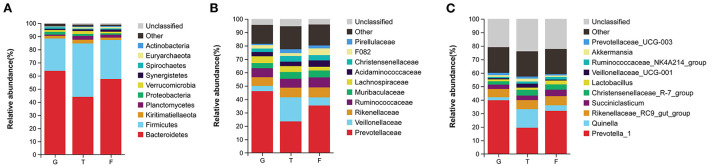
Comparisons of ruminal bacteria in Gangba sheep under different feeding strategies. **(A)** Relative abundances of bacterial communities at the phylum level. **(B)** Relative abundances of bacterial communities at the family level. **(C)** Relative abundances of bacterial communities at the genus level. G, natural grazing; T, semi-grazing with supplementation; F, barn feeding.

**Figure 3:**
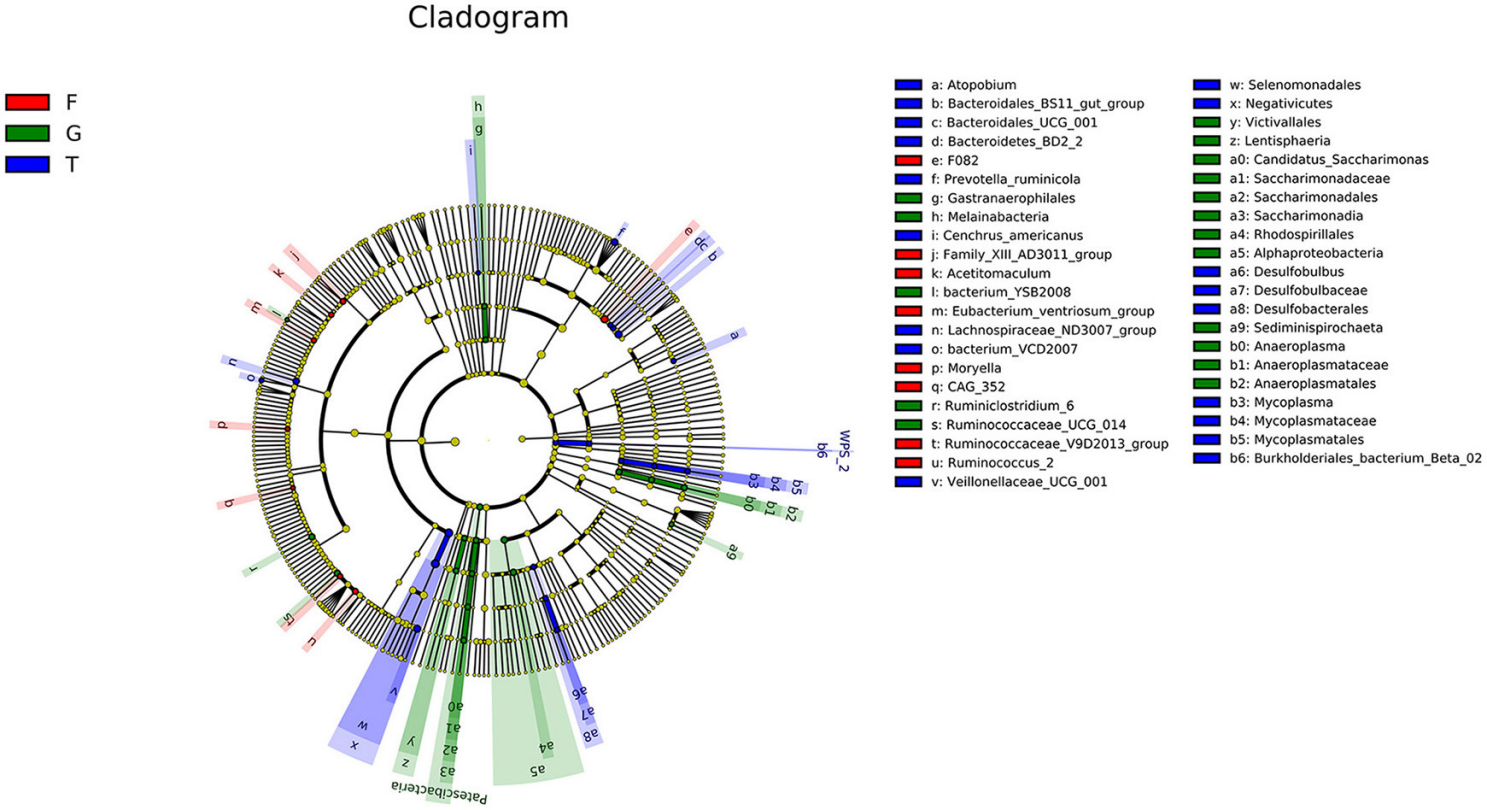
LEFSe (Linear discriminant analysis Effect Size) cladogram comparing microbial communities among the three feeding strategies. Differences are represented by color, indicating the group where taxa are most abundant: red = taxa abundant in F group, green = taxa abundant in G group, blue = taxa abundant in T group. G, natural grazing; T, semi-grazing with supplementation; F, barn feeding.

The microbiota in the rumen were further analyzed. Alpha diversity analyses showed no significant differences in Chao1 richness or Shannon diversity index among the three groups (Figure 1A). In contrast, the beta diversity analyses revealed that the compositions of the rumen microbiota of Gangba sheep under different feeding strategies were significantly different (*P* <0.05; Figure 1B). Taxonomic analysis annotated 24 bacterial phyla at the phylum level. *Bacteroidetes* and *Firmicutes* were the predominant phyla, accounting for 57.55%−63.79% and 24.61%−40.59% of the total sequences, respectively (Figure 2A). *Kiritimatiellaeota, Planctomycetes, Proteobacteria*, and *Verrucomicrobia* represented 1.83%−2.88%, 0.97%−2.86%, 1.09%−2.14%, and 0.99%−1.69% of the total sequences, respectively. At the family level, *Prevotellaceae* (23.46%−46.08%), *Veillonellaceae* (3.91–18.06), *Rikenellaceae* (6.50–7.69%), and *Ruminococcaceae* (6.58%−6.72%) were the dominant families. Other families included *Muribaculaceae* (5.09%−23.10%), *Lachnospiraceae* (2.73%−5.05%), *Acidaminococcaceae* (3.22%−4.82%), *Christensenellaceae* (2.51%−4.25%), and unidentified F082 (2.08%−4.96%; Figure 2B). The relative abundances of *Prevotellaceae, Veillonellaceae*, and F082 were the highest in the G, T and F groups, respectively (Supplementary Figure 1). At the genus level (Figure 2C), *Prevotella*_1, *Quinella, Rikenellaceae*_RC9_gut_group, *Succiniclasticum, Christensenellaceae_*R-7_group, and *Lactobacillus* were the dominant genera in Gangba sheep (relative abundance > 1%). The relative abundances of *Prevotella*_1 and *Succiniclasticum* were the highest (*P* <0.05) in the G and F groups, respectively, while the relative abundances of *Quinella* and *Veillonellaceae_*UCG-001 were the highest (*P* <0.05) in the T group (Supplementary Figure 2). LEfSe was also performed to detect variations in the bacterial taxa composition. As shown in Figure 3, a representative cladogram of the structure of the predominant microbiome revealed the most remarkable differences in taxa among the different feeding strategies. The obtained data demonstrated that 17 clades were most abundant in the G group, 18 clades were most abundant in the T group, and 8 clades were most abundant in the F group. Interestingly, only unidentified F082 was identified as a differential biomarker (LDA score > 4) among the three groups Supplementary Figure 3.

### The correlation between bacterial populations and rumen fermentation profiles

**Figure 4:**
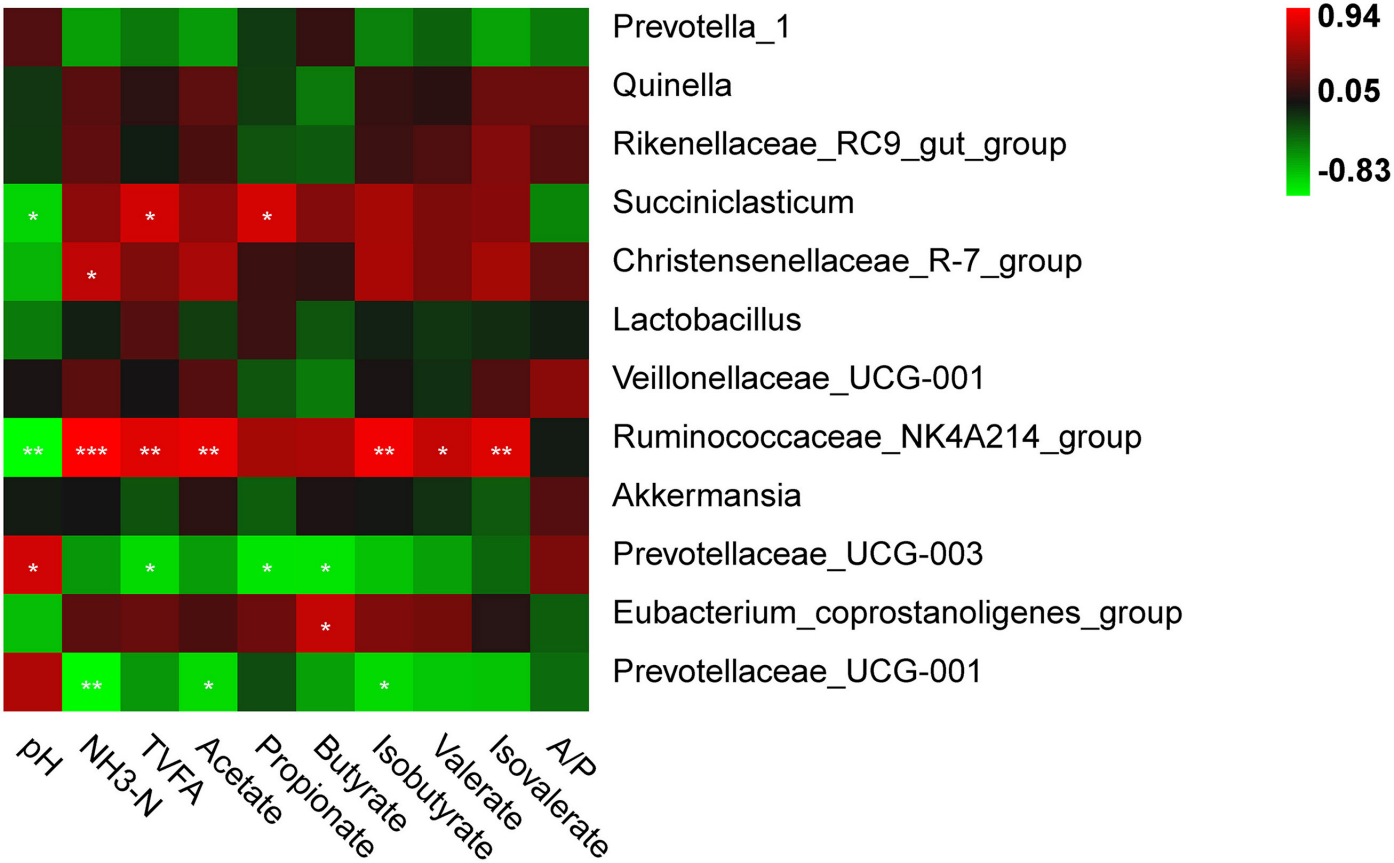
Correlation between the relative abundances of rumen bacteria and fermentation parameters. “*”, “**”, and “***” indicate the significance level at 0.05, 0.01 and 0.001, respectively.

A significant correlation between the relative abundance of rumen bacterial genera and fermentation profiles was observed in Figure 4. The pH value was positively correlated with the relative abundance of the genus *Prevotellaceae_*UCG-003 (*r* = 0.765, *P* = 0.016), and inversely correlated with *Succiniclasticum* abundance (*r* = −0.672, *P* = 0.047). The NH_3_-N concentration was positively correlated with the relative abundances of the genera *Ruminococcaceae_*NK4A214_group (*r* = 0.941, *P* <0.001) and *Christensenellaceae_*R-7_group (*r* = 0.701, *P* = 0.035), and inversely correlated with *Prevotellaceae_*UCG-001 abundance (*r* = −0.812, *P* = 0.007). The TVFA concentration was positively correlated with the relative abundances of *Succiniclasticum* (*r* = 0.772, *P* = 0.015) and *Ruminococcaceae_*NK4A214_group (*r* = 0.819, *P* = 0.007), and inversely correlated with *Prevotellaceae_*UCG-003 (*r* = −0.690, *P* = 0.040). The acetate and isobutyrate concentrations were positively correlated with the relative abundance of the genus *Ruminococcaceae_*NK4A214_group (*r* = 0.857, *P* = 0.003 for acetate; *r* =0.892, *P* = 0.001 for butyrate), while both were negatively correlated with *Prevotellaceae_*UCG-001 abundance (*r* = −0.696, *P* = 0.037 for acetate; *r* = −0.693, *P* = 0.038 for butyrate). The propionate concentration was positively correlated with *Succiniclasticum* (*r* = 0.775, *P* = 0.014) and the butyrate concentration was positively correlated with *Eubacterium_coprostanoligenes_group* (*r* = 0.715, *P* = 0.030), while both were negatively correlated with *Prevotellaceae_*UCG-003 (*r* = −0.739, *P* = 0.023 for propionate; *r* = −0.730, *P* = 0.026 for butyrate). The valerate and isovalerate concentrations were positively associated with *Ruminococcaceae_*NK4A214_group (*r* = 0.713, *P* = 0.031 for valerate; *r* = 0.814, *P* = 0.008 for isovalerate).

### Transcriptome of rumen epithelium and functional gene expression

**Figure 5:**
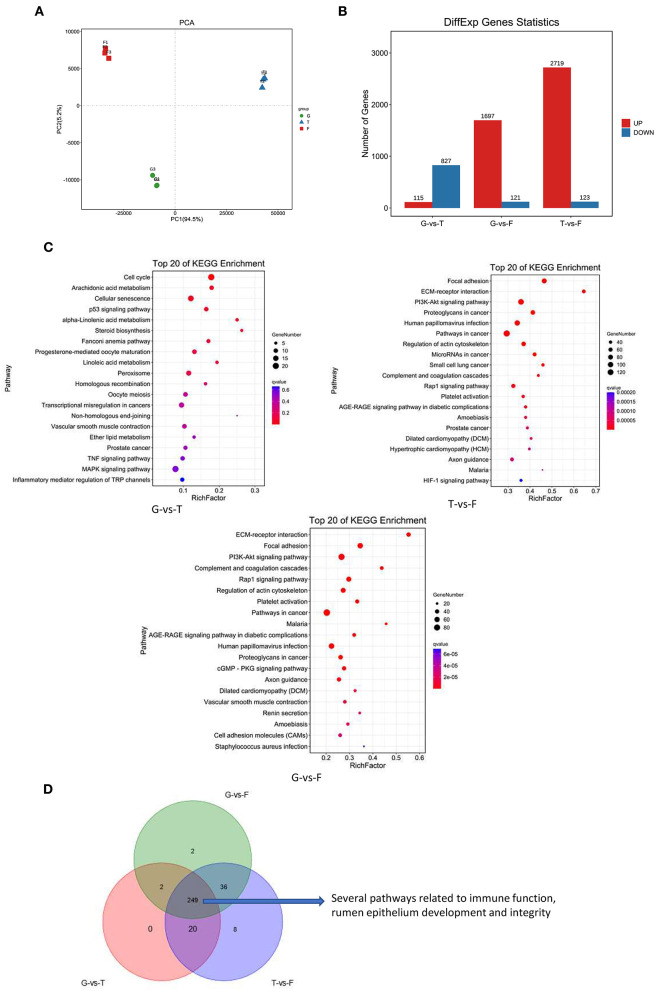
Overview of Gangba sheep ruminal epithelium transcriptome under different feeding strategies. **(A)** PCA plots of transcripts identified by RNA-Seq of Gangba sheep under natural grazing (G), semi-grazing with supplementation (T), and barn feeding (F) conditions**. (B)** Number of individual transcripts significantly up- or down-regulated under different feeding strategies**. (C)** KEGG pathway enrichment analysis of DEGs in the Gangba sheep transcriptome under different feeding strategies. *X*-axis depicts richness factor (Richness factor = DEGs enriched in the pathway/background genes in the pathway). *Y*-axis represents the KEGG pathway terms. The color of each circle represents *q*-value. The area of each circle represents the number of DEGs enriched in this pathway. **(D)** Venn diagram of common and unique pathways presented in the Gangba sheep transcriptome under different feeding strategies.

To identify the global transcriptomic changes that occurred in different feeding strategies, transcriptome data from G, T, and F groups were collected. Principal component analysis of gene expression indicated that different feed strategies induced changes in gene expression (Figure 5A). A total of 942 (115 up and 827 down), 1,818 (1,697 up and 121 down), and 2,842 (2,719 up and 123 down) DEGs were identified between G vs. T, G vs. F, and T vs. F groups, respectively (Figure 5B). Five DEGs were selected for validation by qRT-PCR. These were: phosphoinositide-3-kinase regulatory subunit 3 (*PIK3R3*), integrin subunit alpha 1 (*ITGA1*), collagen type I alpha 2 chain (*COL1A2*), complement C1q C chain (*C1QC*), and myosin light chain kinase (*MYLK*), which are involved in extracellular matrix (ECM)-receptor interactions, the phosphoinositide 3 kinase-protein kinase B (PI3K-Akt) signaling pathway, focal adhesion, complement and coagulation cascades, and regulation of the actin cytoskeleton, respectively. The fold changes of the RNA sequencing results were highly correlated with qRT-PCR results, confirming the reliability of the RNA-Seq data in this study (Supplementary Figure 4).

All DEGs in each group were subjected to KEGG pathway analysis to reveal the major metabolic pathways involved. There were 271, 289, and 313 pathways assigned for G vs. T, G vs. F, and T vs. F groups, respectively, and the top 20 pathways for each feeding strategy are listed in Figure 5C. Pathway analysis identified several DEGs involved in the signaling pathways related to rumen epithelial cell growth and proliferation, immune function, and integrity and barrier function of the rumen epithelium, including ECM-receptor interactions, focal adhesion, PI3K-Akt signaling, complement and coagulation cascades, and regulation of the actin cytoskeleton (Figure 5D).

**Figure 6:**
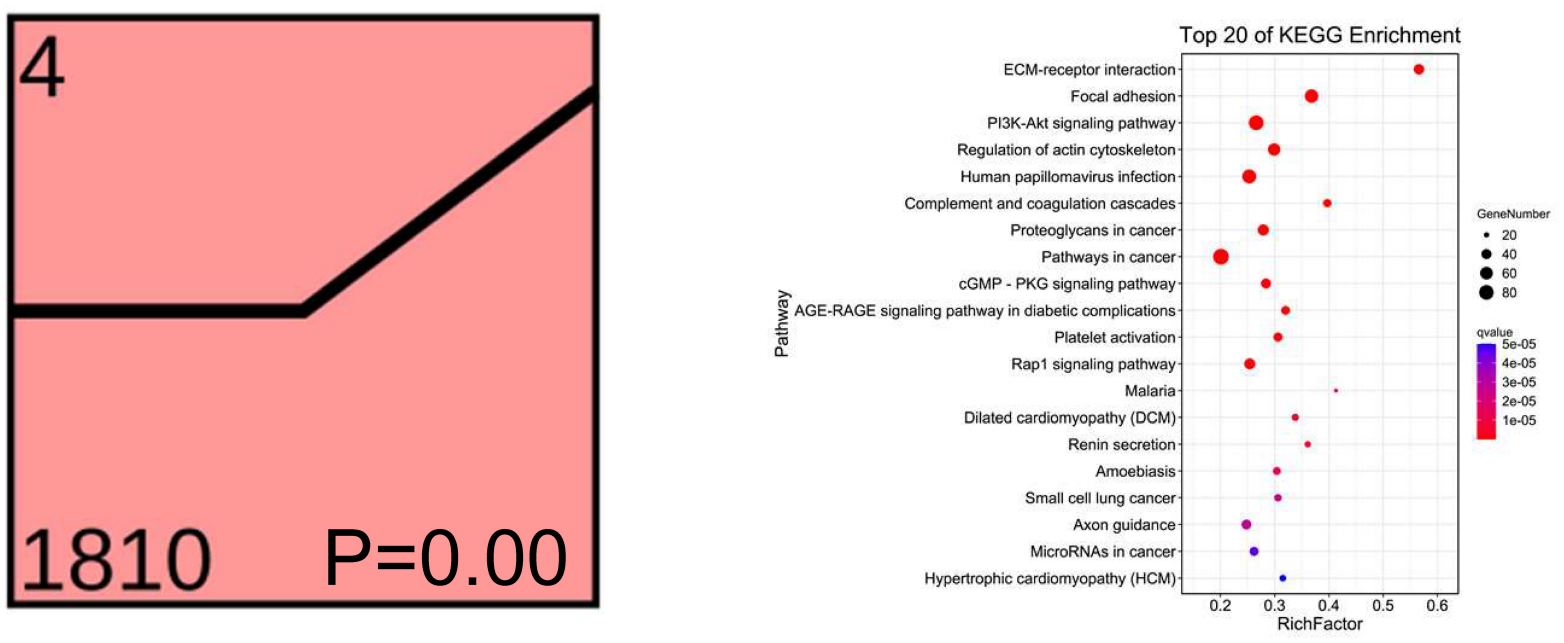
The dynamics of the rumen epithelial transcriptomic analysis of significant DEGs in Profile 4 under different feeding strategies. The top 20 KEGG pathways for Profile 4 are listed on the right. *X*-axis depicts the richness factor (Richness factor = DEGs enriched in the pathway/background genes in the pathway). *Y*-axis represents the KEGG pathway terms. G, natural grazing; T, semi-grazing with supplementation; F, barn feeding.

To understand the dynamics of the rumen epithelial transcriptome under different feeding strategies, Short Time-series Expression Miner analysis (Ernst and Bar-Joseph, 2006) was performed on the total DEGs (Supplementary Figure 5). Profile 4 contained the most transcripts (1,810) and reached the enrichment condition (*P* <0.05; Figure 6). The top 20 enriched KEGG pathways in this profile are listed in Figure 6; many transcripts were involved in controlling immunity, rumen epithelium development, and integrity and barrier functions. These transcripts were not altered in the G and T groups but were increased in the F group, suggesting that these growth-related pathways in ruminant animals were induced by barn feeding rather than feeding strategies involving grazing.

## Discussion

In the present study, an in-depth analysis was conducted to clarify the mechanisms through which feeding strategies impact ruminal homeostasis in a Tibetan local sheep breed, providing the knowledge of an efficient feeding system for grazing animals under harsh environmental conditions.

### Effects of feeding strategies on growth performance of Gangba sheep

Under the same physiological conditions (breed, age, and initial body weight), the G group had lower ADG compared to the T and F groups, due to less DMI and higher ADF content in the diet resulting in the reduced nutrient intake or absorption efficiency. Similar results were also observed in small-tailed Han sheep and Ujumuqin lambs that the final body weight or carcass weight was significantly higher in the confinement group compared to that in the grazing group (Wang et al., 2020; Jin et al., 2022). Our results indicated that the barn feeding strategy can significantly improve the growth performance of Gangba sheep in the F group through the systemic ruminal microbiota-host interactions.

### Effects of feeding strategies on rumen fermentation and bacterial community of Gangba sheep

Previous work has shown that rumen fermentation is closely associated with nutrient supplementation, which resulted in elevated carbohydrate fermentation in the rumen (Raghuvansi et al., 2007; Yang et al., 2018; Liu Y. Z. et al., 2019). In the present study, the significantly increased concentrations of short-chain volatile fatty acids (VFAs), including propionate, butyrate, isobutyrate, valerate, and isovalerate, revealed higher utilization of ruminal energy in the F group. This suggested that greater energy intake and nutrition may be available to support ruminant growth performance when VFAs are absorbed and converted to ruminal nutrients (Orskov, 2012). These nutrients result in different abundances and functions of ruminal microorganisms, further affecting the development and metabolism of host animals (Cui et al., 2020). In this study, the PCoA analysis revealed inherently different ruminal microbiota among sheep subjected to the three different feeding patterns. However, there were no significant differences in the diversity and richness of ruminal microbiota among the three different groups of Gangba sheep. To date, the effects of feeding strategies on ruminal microflora communities in host animals remain a subject of debate. For instance, a previous study found that the alpha diversity of ruminal microbiota in barn-fed yak calves was higher compared to calves from a maternal grazing group (Cui et al., 2020). Furthermore, in tropical rangelands, cattle fed N-based supplements during the dry season revealed no difference in alpha diversity of rumen methanogenic archaea and fungi compared to un-supplemented animals (Franzolin and Wright, 2016). Therefore, other factors such as geography, animal species, and experimental period may contribute to the varied findings.

Tibetan sheep degraded fermentable substrates more efficiently when fed oat hay, as characterized by increased molar proportions of propionate and butyrate due to a shift in microbial populations and VFA-yielding pathways (Zhou et al., 2018). This provided evidence for a correlation between VFA production and the microbial community. In the present study, unidentified F082 was the only differential biomarker at the family level in LEfSe analysis, and its relative abundance was the highest in the F group, compared to the G and T groups. Higher molar proportions of propionate were related to higher relative abundances of unidentified F082 during rumen fermentation (Ma et al., 2020). At the genus level, the microbiota analysis revealed a significant increase in *Succiniclasticum* and *Eubacterium_coprostanoligenes_group* in the F group compared to the G and T groups. These genera were positively correlated with elevated concentrations of propionate and butyrate (Li et al., 2021; Wang B. et al., 2021; Wang Z. et al., 2021). *Succiniclasticum* is a propionate-producing bacterium using succinate (Cao et al., 2020), which is the most important precursor of glucose in ruminants. Similar result was also observed in yak rumen bacteria community that the relative abundance of *Succiniclasticum* was significantly higher in the concentrate group compared to that in the forage group (Liu C. et al., 2019). *Eubacterium_coprostanoligenes_group* has also been shown to produce beneficial SCFAs, especially butyrate, using acetate as a substrate (Wei et al., 2021). Though the relative abundance of *Eubacterium_coprostanoligenes_group* only accounted for approximately 0.5% of the bacterial diversity in the present study, low-abundance taxa did not indicate less significant members of the microbial community. Some species isolated from the rumen microbiome comprised a very small portion thereof but played an important role in consuming the products of primary substrate digestion (Mizrahi et al., 2021). The significantly elevated ruminal VFAs were consistent with the function of significantly enriched genera in the F group, indicating a positive correlation between feed supplementation, ruminal microbiota, and propionate and butyrate production.

### Effects of feeding strategies on ruminal epithelium transcripts of Gangba sheep

Meanwhile, feeding concentrate and oat hay can provide readily fermentable carbohydrates for sufficient microbial production of ruminal VFAs, especially propionate and butyrate, which both serve as energy substrates for animal growth and are required for ruminal epithelium development (Jing et al., 2018; Zhou et al., 2018). Butyrate has been reported to be the most potent stimulator of epithelial proliferation (Blottière et al., 2003). In addition to serving as the primary precursor for ruminant glucogenesis, propionate also acts as a signaling molecule to stimulate the development of the rumen epithelium through up-regulating gene expression of G protein-coupled receptors 41 and 43 (Zhang et al., 2018). Therefore, we explored the mechanism of signal transduction in ruminal epithelium development upon feeding strategies using RNA sequencing. Our results indicated that different feeding strategies, especially barn feeding, could stimulate ruminal epithelium development by regulating the expression of genes involved in cell proliferation-related signal transduction. The PI3K-AKT signaling pathway plays an important role in cell proliferation (Gao et al., 2003). Butyrate promotes epithelial proliferation by acting through the release of growth factors (for example, epidermal growth factor, *EGF*) (Baldwin, 1999). Then, these growth factors induce their cellular responses by binding to their respective cellular membrane receptors (for example, epidermal growth factor receptor, *EGFR*) to initiate a signal transduction cascade involved in cellular proliferation through enhancing expression of protein kinases (for example, the serine/threonine protein kinase, *AKT*). In the current study, up-regulated *EGFR* and *AKT* in the PI3K-AKT signaling pathway in the F group confirmed that butyrate induced ruminal epithelial proliferation to a great extent. ECM-receptor interactions primarily regulate intracellular signal transduction and mediate interactions with cell adhesion receptors to modulate the adhesion, motility, and growth of epithelial cells (Levental et al., 2009). Up-regulation of genes linked to this pathway demonstrated that the barn feeding strategy enhanced the interactions between ECM and membrane receptors and stimulated the activation of intracellular signaling pathway. The focal adhesion pathway is closely associated with cellular proliferation due to the activation of focal adhesion kinase 1 (*PTK2*) which is central to the focal adhesion signaling pathway (Khosravi et al., 2020). As the primary structural protein in the extracellular space, collagen is the most abundant protein in mammals and is encoded by the collagen gene family. The induction of collagen stiffens the ECM, promotes focal adhesions, and enhances PI3K signaling in tumor cell epithelium (Stowers et al., 2016; Wegner et al., 2020). In the present study, the up-regulation of *COL1A, COL4A*, and *COL6A* were commonly observed in the PI3K-AKT, ECM-receptor interactions, and focal adhesion pathways in ruminal epithelium of sheep from the F group, indicating that these three pathways were potentially involved in the regulation of signal transduction for cell proliferation, which was consistent with previous results in other tissues (Qiu et al., 2018). Overall, these findings support the notion that the barn feeding increased propionate and butyrate concentrations by microbial fermentation, promoting ruminal development in growing Gangba sheep. However, the morphology of the ruminal epithelium was not studied and would be an important direction for future studies.

It has been reported that increased VFA-producing microbes could enhance the immune function of barn-fed yak due to the beneficial function of VFAs (Cui et al., 2020). Similar findings were also observed in the present study where genes related to the complement and coagulation cascades were enriched in the F group. These pathways are key constituents of the innate and adaptive immune systems, providing host resistance to potential pathogens (Dunkelberger and Song, 2010). The activation of the complement system cascade includes classical, alternative, and lectin pathways. Of these, the classical pathway is a major component of the innate immune that defends against pathogens and altered self when triggered by the multiprotein complex C1 (Bally et al., 2009). The C1 complex is assembled from the combination of recognition proteins (C1q, C1r and C1s), which can bind and activate the downstream substrate C4 to initiate the reaction cascade (Almitairi et al., 2018). As an integral part of the complement system, complement component C3 (C3) plays an essential role in innate defense where its covalent attachment to pathogens is one of the most critical steps in complement activation (Lamping et al., 2000). Complement component C7 (C7) is one of the complement proteins that facilitates cell death upon activation of either the classical or the alternative pathway (Zarkadis et al., 2005). In this study, the above DEGs relating to the complement and coagulation cascade pathways were all upregulated in the F group, suggesting that the rich supplementation of carbohydrates from both fibrous and non-fibrous sources from barn feeding strategy was helpful to improve immune functions in Gangba sheep and that this resulted from increased VFAs, especially propionate and butyrate, in the rumen (Cui et al., 2020).

In addition to immune function, the ruminal epithelium plays a crucial role in epithelial permeability in animals (Klevenhusen et al., 2013). Though there was no greater expression of genes involved in SCFA absorption in the rumens of barn-fed animals, there was clear evidence demonstrating differences in the expression of genes associated with paracellular permeability, which could influence the diffusion of SCFAs and other nutrients (Kong et al., 2016). In this study, regulation of the actin cytoskeleton was a pathway that was enriched and upregulated in the epithelia of the barn feeding group. The regulation of the actin cytoskeleton is vital to multiple biological processes such as the maintenance of tissue integrity and paracellular permeability (Rodgers and Fanning, 2011). For example, the DEGs involved in this pathway included myosin light chain (*MLC*), a regulator of the perijunctional actomyosin ring (PAMR) which controls epithelial tight junctions (TJ) (Turner et al., 1997). Additionally, *MLC* requires activation by a key regulator, myosin light chain kinase (*MLCK*), in order to regulate PAMR contraction, thereby supporting the regulation of epithelial permeability (Wu et al., 2010). Furthermore, the barn feeding group displayed upregulation of several DEGs encoding Ras GTPase-activating-like proteins IQGAP1, 2 and 3 (*IQGAP1, IQGAP2 and IQGAP3*), and Rho guanine nucleotide exchange factors 1 and 12 (*ARHGEF1* and *ARHGEF12*). These proteins are involved in the remodeling of epithelial adherens junctions and tight junctions (Shaifta et al., 2018). Overall, the increased expression of genes related to the regulation of the actin cytoskeleton pathway suggests greater cell dynamic remodeling within the epithelium of the barn feeding group. These may result in small gaps between cells leading to higher paracellular permeability for the absorption of nutrients, especially SCFAs. Therefore, the supplementation of concentrate and oat hay in the barn feeding group may facilitate greater nutrient absorption through increased paracellular permeability, thus satisfying the elevated nutritional requirement for growth. Similar results were also observed in the rumen epithelium of highly efficient steers, which had higher expression of genes related to cytoskeletal dynamics compared with inefficient steers (Kong et al., 2016). Additional studies are still needed to measure the intercellular space and nutrient absorption in the ruminal epithelium to determine whether there were true differences among different feeding strategies.

## Conclusion

In summary, our study shows that barn feeding with concentrate and oat hay supplementation is beneficial to ruminal epithelium development and its absorption and immune functions. Thus, barn feeding enhanced the growth of Gangba sheep by taking advantage of the increased species diversity and abundance of different microbes that could generate more VFAs, especially propionate and butyrate, using different carbon sources from both fibrous and non-fibrous carbohydrates. Therefore, the barn feeding strategy for local sheep is recommended to improve growth performance under harsh conditions in the Tibetan alpine pastoral livestock systems.

## Data availability statement

The sequencing data were deposited in the Sequence Read Archive of the National Center for Biotechnology Information database with accession project numbers PRJNA761328 and PRJNA863596.

## Ethics statement

The animal study was reviewed and approved by the Committee on Experimental Animal Management of the Chinese Academy of Agricultural Sciences.

## Author contributions

ZJ, DZ, and ZX developed and framed the research questions. ZX and ZJ analyzed transcriptome, microbiome data, and involved in revising the manuscript. TN was involved in the analysis of animal growth performance and rumen fermentation data. GJ, LC, and PB were involved in sample collection. ZJ and DZ drafted the manuscript. All authors contributed to the article and approved the submitted version.

## Funding

The work was funded by the Major Science and Technology Program of Tibet (XZ2021ZD0001N), the Key Research and Development Program of Tibet (XZ202001ZY0037N), the Special Funds for the Local Science and Technology Development of the Central Government (2020ZY0021), and Natural Science Foundation of Inner Mongolia (2022LHMS03005) for the financial support to the research in our laboratory.

## Conflict of interest

The authors declare that the research was conducted in the absence of any commercial or financial relationships that could be construed as a potential conflict of interest.

## Publisher's note

All claims expressed in this article are solely those of the authors and do not necessarily represent those of their affiliated organizations, or those of the publisher, the editors and the reviewers. Any product that may be evaluated in this article, or claim that may be made by its manufacturer, is not guaranteed or endorsed by the publisher.

## Supplementary material

Supplementary Table 1 Primer sequences of the target genes for qPCR analysis.

Supplementary Figure 1 Effects of feeding strategies on the rumen bacterial composition at the family level in Gangba sheep. The bar graph shows the significantly-changed bacteria at the family level. Values are means ± SD. Bars with different small letter subscripts are significantly different (*P* <0.05). G, natural grazing; T, semi-grazing with supplementation; F, barn feeding.

Supplementary Figure 2 Effects of feeding strategies on the rumen bacterial composition at the genus level in Gangba sheep. The bar graph shows the significantly-changed bacteria at the genus level. Values are means ± SD. Bars with different small letter subscripts are significantly different (*P* <0.05). G, natural grazing; T, semi-grazing with supplementation; F, barn feeding.

Supplementary Figure 3 LDA value distribution histogram. LDA value >4, and the length of the bar chart represents the influence of different species. G, natural grazing; T, semi-grazing with supplementation; F, barn feeding.

Supplementary Figure 4 qRT-PCR validation of differentially-expressed genes from the ruminal epithelium of Gangba sheep. β-actin was used as an internal control, and data are presented as fold change (*n* = 6 per group).

Supplementary Figure 5 The dynamics of the rumen epithelial transcriptomic analysis of significant DEGs under different feeding strategies. Profiles ordered based on the number of genes assigned.
