# Supplementary material for: Different feeding strategies can affect growth performance and rumen functions in Gangba sheep as revealed by integrated transcriptome and microbiome analyses
Source: Front Microbiol. 2022 Aug 24;13:908326. doi: 10.3389/fmicb.2022.908326 (PMC9449551; doi:10.3389/fmicb.2022.908326)
Supplement: Supplementary Table 1 — Primer sequences of the target genes for qPCR analysis. [file Table_1.DOCX]

**Table 1** Primer sequences of the target genes for qPCR analysis

| Gene name | Primer sequence |
| --- | --- |
| *ACTB* | forward: AGATTGGCCTCGTGCGATTC  reverse: CATGGTTGCTAAGGGCAGGA |
| *PIK3R3* | forward: GCGCGATGTACAATACGGTG  reverse: GCTTTGGTGGAAGAGCTGGA |
| *ITGA1* | forward: AGTGCTGTGGAAGATTGGATT  reverse: TGAGGCAAACCTGAGGATTATTGA |
| *COL1A2* | forward: CCTGGACCAATGGGGTTGAT  reverse: CAGGTCGTCCAGGTTTTCCA |
| *C1QC* | forward: CACCTGTCCAGCTCCTTCC  reverse: ATCCCATAGCAGTGTGTGCC |
| *MYLK* | forward: AGGACTGCACTGTCGTTGAG  reverse: ACTGGATGGGCTGTCCATTG |
