# Supplementary figures and images for: Different feeding strategies can affect growth performance and rumen functions in Gangba sheep as revealed by integrated transcriptome and microbiome analyses
Source: Front Microbiol. 2022 Aug 24;13:908326. doi: 10.3389/fmicb.2022.908326 (PMC9449551; doi:10.3389/fmicb.2022.908326)

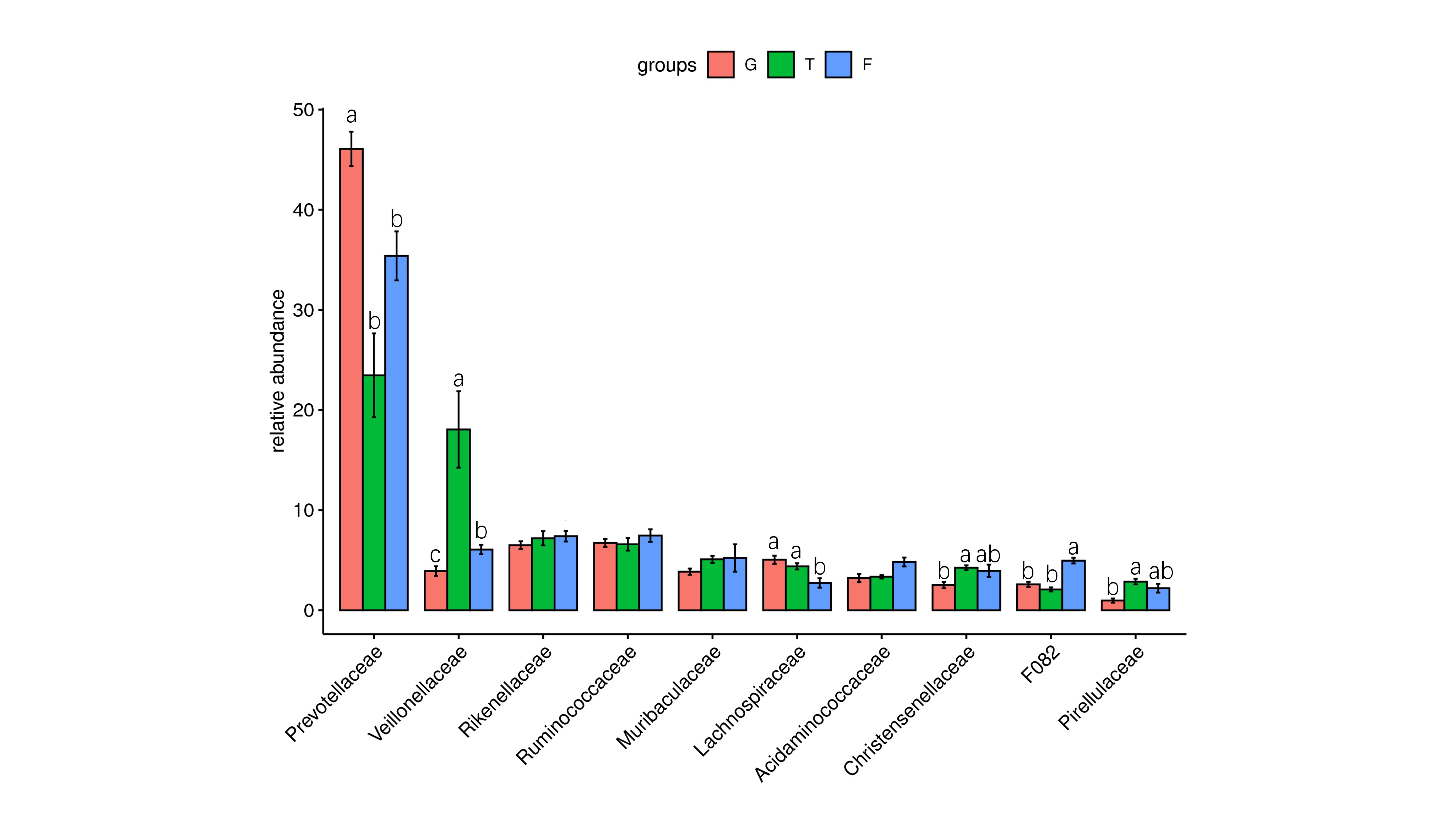

Supplement: Supplementary Figure 1 — Effects of feeding strategies on the rumen bacterial composition at the family level in Gangba sheep. The bar graph shows the significantly-changed bacteria at the family level. Values are means ± SD. Bars with different small letter subscripts are significantly different (P <0.05). G, natural grazing; T, semi-grazing with supplementation; F, barn feeding. [file Image_1.TIFF]

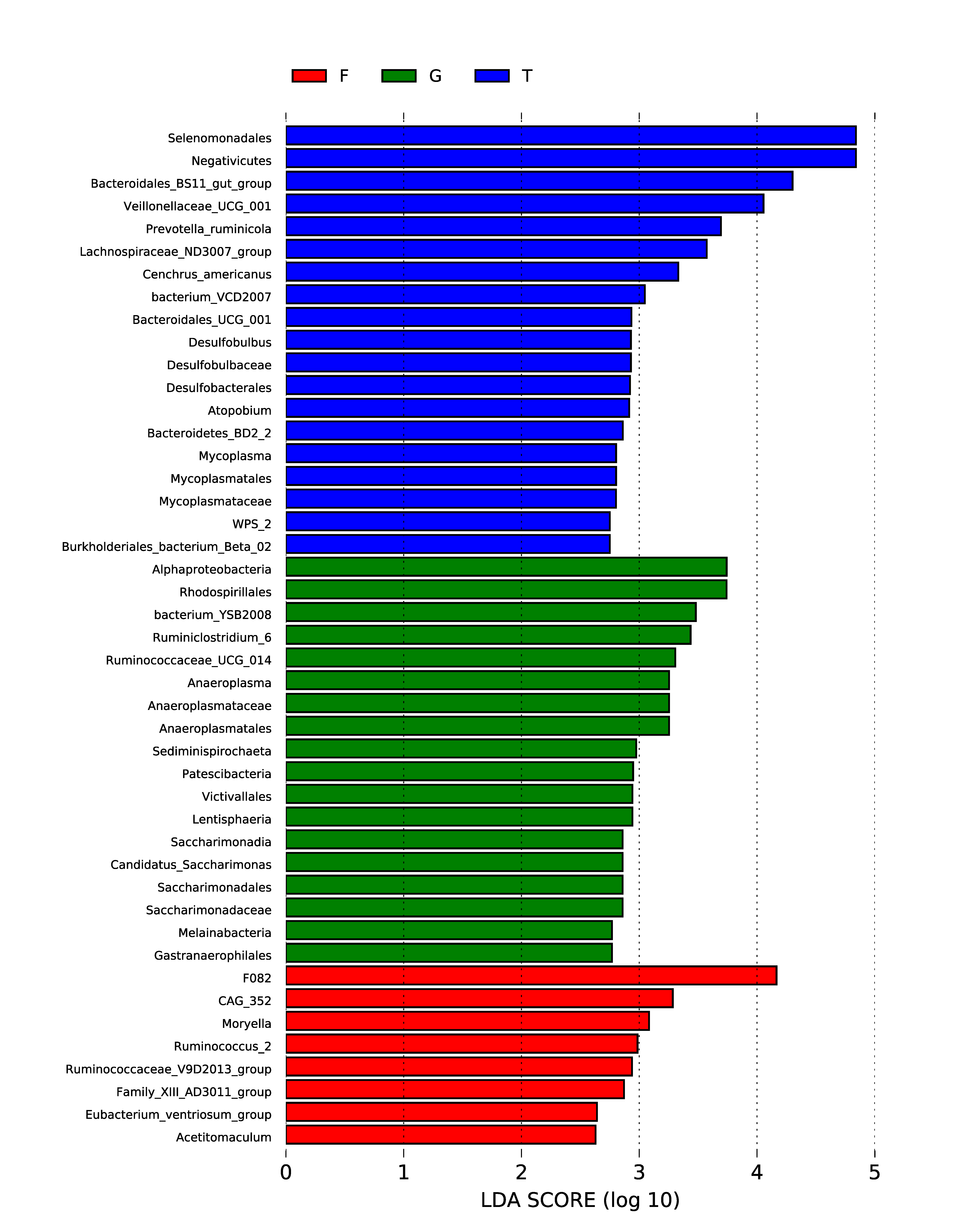

Supplement: Supplementary Figure 3 — LDA value distribution histogram. LDA value >4, and the length of the bar chart represents the influence of different species. G, natural grazing; T, semi-grazing with supplementation; F, barn feeding. [file Image_3.TIFF]

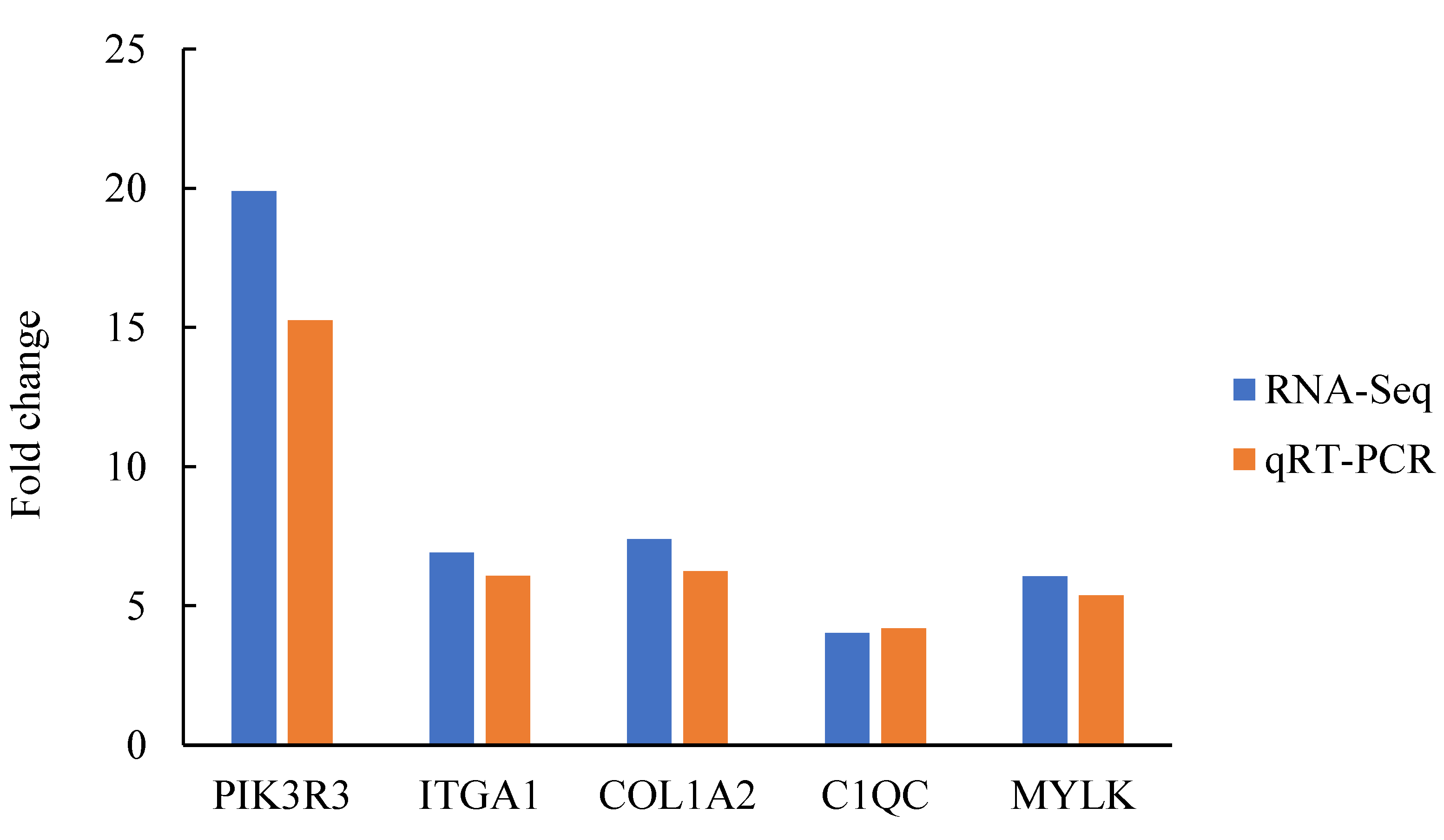

Supplement: Supplementary Figure 4 — qRT-PCR validation of differentially-expressed genes from the ruminal epithelium of Gangba sheep. β-actin was used as an internal control, and data are presented as fold change (n = 6 per group). [file Image_4.TIFF]

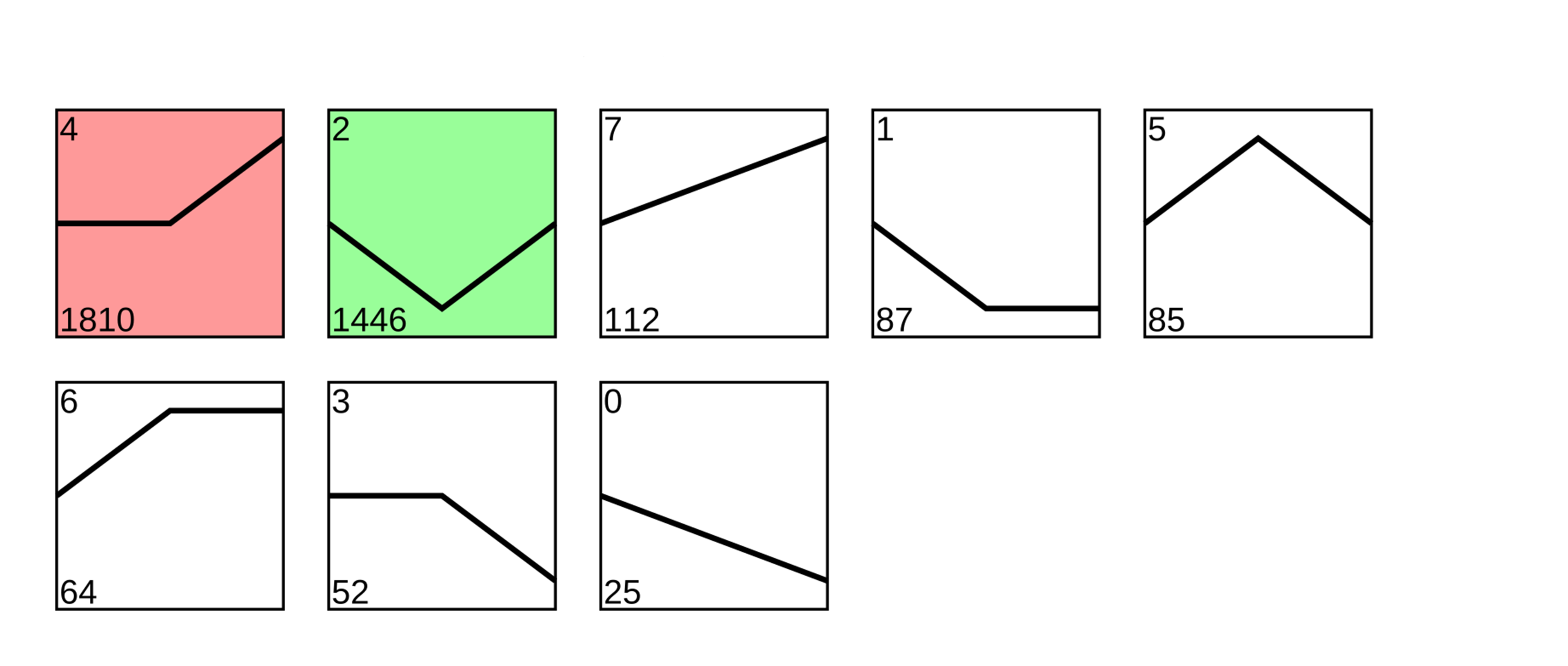

Supplement: Supplementary Figure 5 — The dynamics of the rumen epithelial transcriptomic analysis of significant DEGs under different feeding strategies. Profiles ordered based on the number of genes assigned. [file Image_5.TIF]
